# A Rare Case of Low-Grade Serous Cystadenocarcinoma of the Ovary in a Seventeen-Year-Old Woman

**DOI:** 10.7759/cureus.28086

**Published:** 2022-08-16

**Authors:** John Rickards, Byron A Brooks, Ana Rivera-Betancourt, Douglas Velasquez

**Affiliations:** 1 Medicine, Trinity School of Medicine, Rishibi, VCT; 2 Obstetrics and Gynecology, Macon OBGyn Associates, Macon, USA; 3 Research, Trinity School of Medicine, Rishibi, VCT

**Keywords:** serous cystadenoma, obgyn oncology, gyn pathology, atypical presentation, abnormal vaginal bleeding

## Abstract

Epithelial ovarian cancer is the most prevalent gynecological malignancy leading to mortality in adults but is an infrequent finding in children and adolescents. Most gynecological tumors found in females younger than age 20 are of germ cell nature. There are very few cases of epithelial ovarian neoplasms reported in the young female population. We report a case of low-grade serous ovarian neoplasm in a 17-year-old Caucasian female.

## Introduction

Ovarian cancer is responsible for the majority of deaths from gynecological malignancies in the United States, with a five-year survival rate of less than 45% [1,2]. In adults, more than half of all ovarian tumors are of an epithelial origin, while in children and adolescent populations, germ cell tumors dominate these statistics [3]. Advancing age and genetic predisposition are known risk factors associated with ovarian cancer. However, its exact pathophysiology is still not fully understood [4,5]. Early diagnosis and intervention with surgical and chemotherapeutic methods have proven to effectively treat this condition. However, the non-specificity of the common presenting symptoms makes diagnosis challenging, especially in populations with documented low incidence [5]. In this case report, we describe a 17-year-old Caucasian female who initially presented due to recurrent abnormal uterine bleeding and abdominal pain. Following surgery and pathological analysis, she was definitively diagnosed with an epithelial ovarian neoplasm.

## Case presentation

This case reviews the management of a 17-year-old Caucasian female who presented for surgical consultation due to a 13-month history of prolonged abnormal uterine bleeding and dysmenorrhea. The patient continually had uterine bleeding between her menses that reportedly appeared more voluminous than her menses at times. She also reported pelvic pain without radiation, rated at 7/10 in severity prior to and after the commencement of her menses. It was noted that this pain had increased over the week prior to her visit. Visceral symptoms such as nausea and vomiting were also noted and coincided with the increased severity of her pelvic pain. At the time of this visit, the patient had an etonogestrel 68 mg implant in place due to previous complaints of abnormal uterine bleeding. She reported minimal relief in her symptoms after implantation. She had also previously been prescribed levonorgestrel-ethinyl estradiol PO once a day, a combined oral contraceptive therapy, which was also unsuccessful in terminating her symptoms. Elagolix 150 mg PO once a day was also previously prescribed, but she was unable to tolerate it, reporting severe headache and nausea.

A physical examination revealed unremarkable cardiovascular and respiratory system assessments. An abdominal examination revealed no ascites, tenderness, guarding, or rigidity. A gynecological examination revealed a normal external vagina and visualization of the cervix was made with no noted abnormalities. Past medical history was also assessed and no significant illness was noted. Her family history was significant only for hypertension on her mother's side of the family. It was, however, negative for any cancer, chronic illness, or gynecological conditions.

**Figure 1:**
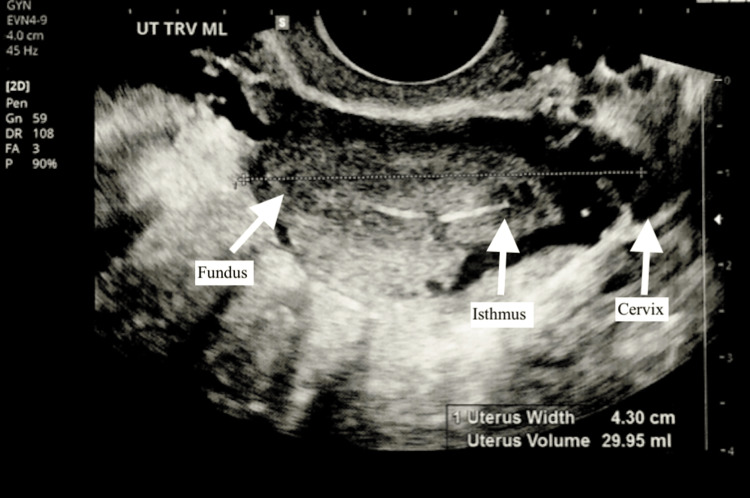
Transvaginal ultrasound of midsagittal view of uterus with arrows labeling fundus, isthmus, and cervix

The symptoms of abnormal uterine bleeding and dysmenorrhea were discussed with the patient, and a presumptive diagnosis of endometriosis was made. The patient expressed her desire for a definitive diagnosis and treatment, and therefore, diagnostic laparoscopy was scheduled to confirm and treat her suspected endometriosis. An ultrasound (Figure 1) was performed during the visit and reviewed with the patient. It revealed an anteverted uterus with visualization of both ovaries with follicles and normal blood flow.

Laparoscopic surgery was performed, resulting in the removal of suspected implants of endometriosis via peritoneal stripping at the posterior cul-de-sac and lysis of filmy adhesions to the ileocecal junction and appendix. During the surgery, the ovaries, fallopian tubes, uterus, and peritoneum were found to be visually normal. The specimens removed were fixed with formalin and paraffin-embedded, following which they were sent for pathological analysis to confirm their histological construct and definitively diagnose the patient.

The pathological analysis demonstrated serous glandular proliferation within an inflamed and hemorrhagic reactive-appearing fibrous stroma. Some glands were associated with cuffs of CD10+ endometrial-type stroma, suggestive of endometriosis. However, the majority of glands lacked an associated stromal cuff and showed numerous psammomatous calcifications and architectural complexity with focal papillary configuration. No increased mitotic activity, necrosis, or high-grade nuclear features were seen. These unusual findings led to the sample being referred to the University of Michigan for expert consultation. This analysis resulted in findings consistent with a diagnosis of papillary serous neoplasm implant.

In light of this finding, and the unremarkable anatomical findings noted during the laparoscopic procedure, it was decided to seek further extra-departmental consultation from the Cleveland Clinic, Ohio. This analysis returned findings consistent with a low-grade serous neoplasm. These pathological findings led to the establishment of a differential diagnosis of a non-invasive implant of a serous borderline tumor of ovarian origin, a primary peritoneal serous borderline tumor, and less likely, papillary endosalpingiosis mimicking a serous neoplasm.

This diagnosis was discussed with the patient and a referral to an oncologist was made for continuity of treatment following this new finding. The patient was then seen after the procedure, where her menorrhagia and dysmenorrhea were evaluated for improvement. Genetic testing was also performed due to the rarity of this diagnosis within her age group as well as her unremarkable family history. This testing revealed negative results for BRCA1, BRCA2, or any other notable mutations. A discussion regarding mature oocyte cryopreservation was also conducted. On this visit, the patient noted a reduction in her pain level, describing it as 2/10 in severity. She still experienced mild bleeding between periods. Upon discussion, a progestin intrauterine device was implanted and her etonogestrel implant was removed. The patient proceeded with treatment under the supervision of her oncologist.

## Discussion

Globally, ovarian cancer accounts for over 200,000 deaths annually, making it the eighth most common cause of cancer-related death in women. Among the types of ovarian cancer that exist, epithelial-related neoplasms account for over 90%, with the serous subtype being the most common. Furthermore, non-specific symptoms lead to misidentification of this diagnosis and possible attribution of these symptoms to other disease processes. Increasing age correlates with an increased incidence of disease, and therefore, postmenopausal women are most affected [1,4].

The pathogenesis of epithelial ovarian cancer is still a highly debated topic. The most widely accepted theory describes cellular DNA damage, due to repeated physical trauma of surface epithelial cells during ovulation, as the catalyst for developing ovarian tumors. This theory aligns with the epidemiological data for ovarian cancer as well as the known risk factors of early menarche and late-onset menopause. In 2004, a theory brought forward by Kurman and Shih grouped ovarian cancers into two categories according to their histological, clinical, and genetic makeup. This "two-pathway theory" describes type 1 ovarian tumors as being associated with K-RAS and BRAF mutations, as well as being characteristically more benign and growing more slowly. Contrastingly, type 2 ovarian tumors were described as being more aggressive and genetically unstable. They were said to be associated with BRCA1/2 and TP53 mutations [5]. Our patient's findings most closely align with a type 1 ovarian tumor due to its benign, low-grade nature.

Our case is remarkable as it features a 17-year-old female who was definitively diagnosed with a low-grade serous neoplasm. Among this population, germ cell tumors are far more common and the occurrence of an epithelial ovarian neoplasm is a very rare phenomenon, with the incidence estimated at 2.6 cases per 100,000 girls per year [3,6]. Family history and the presence of genetic mutations are noted as some of the strongest risk factors for the development of ovarian cancer [1]. Noteworthy is the fact that this patient’s genetic testing revealed no abnormalities and her family history was not significant for any cancer, gynecological, or other related condition. 

Abnormal uterine bleeding and dysmenorrhea in a female under the age of 20 should lead to prompt evaluation with an ovarian neoplasm included as a possible differential diagnosis. However, in this population, other diagnoses such as endometriosis have a similar presentation and are far more likely. Due to the risk associated with an invasive procedure, the use of pharmacological treatments such as oral contraceptive pills offers a viable alternative for the resolution of symptoms, especially when the physical exam is normal, imaging is unremarkable, and alarming symptoms are absent, as seen in this patient. A definitive diagnosis still relies on laparoscopic surgery, and in the event of treatment failure, this is the preferred next step in management. If an ovarian tumor is suspected, preoperative tumor markers such as CA125 may assist with the diagnosis but have been proven to be unreliable and non-specific. Screening methods, including imagining and physical examination, have not led to any reduction in cancer incidence [7].

Most of these tumors, especially when low grade, may be treated with a conservative approach that maintains fertility, which is especially favorable in this young population [7]. A dual therapeutic approach inclusive of surgical debulking and chemotherapy with the use of cisplatin and paclitaxel is the standard treatment plan for this diagnosis. In young patients such as ours, emphasis is placed on preserving fertility, and thus a unilateral salpingo-oophorectomy is preferred once they are good candidates. However, a surgical approach comprising a hysterectomy or bilateral salpingo-oophorectomy (BSO) has been shown to have better outcomes in more advanced disease states [1]. Primary prevention through prophylactic surgery has also been recommended for high-risk populations such as those with BRCA1/2 mutations.

Our patient is currently undergoing chemotherapeutic treatment and efforts are being made to preserve her fertility. Importantly, the documentation of such a clinical phenomenon is worthwhile reporting as it broadens clinicians' management of similar presentations to include the possibility of such an occurrence.

## Conclusions

We presented an uncommon case of an epithelial ovarian neoplasm in a teenage patient. This is a rarity and often excluded from possible differential diagnoses within this population due to its unlikeliness and the non-specificity of the common presenting symptoms. Furthermore, in younger populations, such as this patient, the preservation of fertility is paramount and diagnostic accuracy is vital. With early diagnosis and intervention being a pillar of this gynecological condition, highlighting the possible incidence of such a case is significant. This case highlights the need to perform thorough clinical, radiological, pathological, and laboratory examinations on all gynecological patients irrespective of age.
